# Molecular Characterization and Expression Analysis of Putative Class C (Glutamate Family) G Protein-Coupled Receptors in Ascidian *Styela clava*

**DOI:** 10.3390/biology11050782

**Published:** 2022-05-20

**Authors:** Jin Zhang, Bo Dong, Likun Yang

**Affiliations:** 1 Sars-Fang Centre, MoE Key Laboratory of Marine Genetics and Breeding, College of Marine Life Sciences, Ocean University of China, Qingdao 266003, China; zhangjincoolcool@163.com; 2 Laboratory for Marine Biology and Biotechnology, Qingdao National Laboratory for Marine Science and Technology, Qingdao 266237, China; 3 Institute of Evolution & Marine Biodiversity, Ocean University of China, Qingdao 266003, China

**Keywords:** *Styela clava*, Class C G protein-coupled receptor, neurotransmission, conserved domains, expression pattern

## Abstract

**Simple Summary:**

Ascidians, known as the closest invertebrate relative to the vertebrate group, have a biphasic life cycle including the larval and sessile adult stages with strong adaptability to diverse environments. The nervous system of ascidians plays an essential role in its adaption to the external environment, but the molecular mechanisms underlying this process still need to be further clarified. The Class C G protein-coupled receptors (GPCRs) are a group of cell surface sensors for neurotransmitters and external chemicals, playing important functions in neurotransmission. We systematically characterized the putative Class C GPCRs in an important invasive ascidian species *Styela clava* and then analyzed their expression levels during different developmental stages and distribution in swimming larvae and multiple tissues of the adults. Our study suggests that *S. clava* Class C GPCRs potentially function as important molecules during neurotransmission related to physiological and morphogenetic changes in larvae and adults.

**Abstract:**

In this study, we performed the genome-wide domain analysis and sequence alignment on the genome of *Styela clava*, and obtained a repertoire of 204 putative GPCRs, which exhibited a highly reduced gene number compared to vertebrates and cephalochordates. In this repertoire, six Class C GPCRs, including four metabotropic glutamate receptors (Sc-GRMs), one calcium-sensing receptor (Sc-CaSR), and one gamma-aminobutyric acid (GABA) type B receptor 2-like (Sc-GABA_B_R2-like) were identified, with the absence of type 1 taste and vomeronasal receptors. All the Sc-GRMs and Sc-CaSR contained the typical “Venus flytrap” and cysteine-rich domains required for ligand binding and subsequent propagation of conformational changes. In swimming larvae, *Sc-grm3* and *Sc-casr* were mainly expressed at the junction of the sensory vesicle and tail nerve cord while the transcripts of *Sc-grm4*, *Sc-grm7a*, and *Sc-grm7b* appeared at the anterior trunk, which suggested their important functions in neurotransmission. The high expression of these Class C receptors at tail-regression and metamorphic juvenile stages hinted at their potential involvement in regulating metamorphosis. In adults, the transcripts were highly expressed in several peripheral tissues, raising the possibility that *S. clava* Class C GPCRs might function as neurotransmission modulators peripherally after metamorphosis. Our study systematically characterized the ancestral chordate Class C GPCRs to provide insights into the origin and evolution of these receptors in chordates and their roles in regulating physiological and morphogenetic changes relevant to the development and environmental adaption.

## 1. Introduction

Ascidians, belonging to the phylum Chordata tunicates, are the closest extant relatives of vertebrates, occupying a critical evolutionary position between invertebrates and vertebrates [1]. Like many marine invertebrates, ascidians have a biphasic life cycle, undergoing the metamorphosis from the larval to the sessile juvenile/adult stage [2]. As ascidians own several features, such as transparent and vertebrate-like bodies at the early developmental stage, rapid development processes, and compact genome size, they are suitable models for exploring the evolutionary origins of the vertebrate genes and the regulatory mechanisms underlying the developmental process [1]. In addition to their significance in terms of evolutionary and developmental biology, many species of ascidians, such as *Ciona*, *Botryloides*, *Styela*, *Eudistoma*, and *Botrylus*, are also known as fouling organisms with strong adaptability to diverse environmental conditions, causing many biofouling problems and serious economic losses in marine ecosystems, such as aquaculture, maritime industries, and water conservancy projects [3,4].

It has been known that ascidians contain both the central nervous system (CNS) and peripheral nervous system (PNS) with the essential functions of sensing external stimuli, transmitting a series of molecular and cellular responses, and generating numerous rapid physiological and morphogenetic changes to adapt to the external environment (e.g., gravity and light perception [5], locomotion [6], and metamorphosis [7,8]). The CNS of the ascidian larva, along the anterior-posterior axis, structurally consists of four parts, a sensory vesicle, neck, trunk ganglion, and caudal tail nerve cord, sharing basic homologies with the vertebrate CNS [9,10]. To date, although some signaling pathways associated with the nervous system in the regulation of larval swimming behaviors and metamorphosis have been revealed [11,12,13,14], the regulatory mechanisms at molecular levels still need to be further clarified.

The G protein-coupled receptors (GPCRs) are the most common cell surface protein superfamily that plays a central role in cell signaling as receptors for several neurotransmitters, mediators, hormones, and neuropeptides, connecting the extracellular signals with intracellular signaling [15,16]. Activated GPCRs can trigger signal transduction to influence intracellular secondary messenger levels and protein kinase activation in G protein-dependent and -independent manners [17,18]. Based on sequence homology and functional similarity, GPCRs are divided into six subfamilies including rhodopsin-like (Class A), secretin-like (Class B), glutamate (Class C), fungal mating pheromone (Class D), cAMP (Class E), and frizzled (Class F) GPCRs [19]. Among these subfamilies, Class C GPCRs are a small group of receptors that structurally possess a long N-terminal extracellular domain inclusive of the “Venus flytrap” domain (VFD) that encloses the orthosteric binding site responsible for ligand recognition and binding. By obligatorily forming homodimers/heterodimers, these receptors trigger intracellular signaling to modulate the function of various effectors, such as enzymes, ion channels, and transcription factors [20,21,22]. 

In vertebrates, Class C GPCRs consist of metabotropic glutamate receptors (GRMs), gamma-aminobutyric acid (GABA) type B receptors (GABA_B_Rs), extracellular calcium-sensing receptor (CaSR), type 1 taste receptors (TAS1Rs), vomeronasal receptors (VRs), and several orphan receptors. The GRMs are receptors for excitatory neurotransmitter L-glutamate and GABA_B_Rs are receptors for the inhibitory neurotransmitter GABA, which are well known to be indispensable in the modulation of synaptic transmission and neuronal excitability throughout the nervous system [23]. The CaSR has been known as a critical sensor for extracellular Ca^2+^ levels and regulates the calcium balance in the cytoplasm, which is implicated in synaptic plasticity and neurotransmission [24]. The TAS1Rs are receptors for recognizing diverse natural and synthetic sweeteners as well as umami taste stimuli [25]. The VRs are a group of olfactory receptors that are abundant in rodents and putatively function as receptors for pheromones [26].

Until now, the Class C GPCRs have been extensively investigated in vertebrates. However, the analyses of this subfamily in other metazoans have not been well performed and their physiological relevance is largely unknown. In the ascidian *Ciona robusta*, Class C GPCRs including GRMs, GABA_B_Rs, and CaSR have been identified through genomic and transcriptomic analysis [27]. It has been shown that the transcripts of *C. robusta* GABA_B_R1 and GABA_B_R2 appeared at the tail-bud stage in a few visceral ganglion precursor cells, and were specifically expressed in the sensory vesicle and visceral ganglion of the larva [28], supporting their important roles in neurotransmission and its related physiological functions, such as locomotion [29] and induction of larval metamorphosis via the neuropeptide signaling [13]. However, other members of this subfamily have not been well characterized. The systematic analysis of this subfamily is still required to provide a theoretical basis for further investigating their physiological functions.

In the present study, we systematically identified and characterized Class C GPCRs in the ascidian *Styela clava*, a native ascidian species in the northwestern region of the Pacific and is now a predominant ascidian species in the coastal area of China [12,30,31]. We then conducted phylogenetic analysis, sequence analysis, and functional domain prediction of *S. clava* Class C GPCRs. Moreover, we investigated their expression levels during different developmental stages and distribution in swimming larva and multiple tissues of the adults. The present study will provide insights into the origin and evolution of Class C GPCRs in chordates and their potential roles in the regulation of physiological and morphogenetic changes, laying a theoretical foundation for further functional investigations.

## 2. Materials and Methods

### 2.1. Identification and Classification of S. clava GPCRs

We started with a comprehensive analysis using both sequence alignment (BLASTP) and genome-wide domain analysis. The BLASTP based on sequence homology is a common method for the identification of putative GPCRs. We collected protein sequences of GPCRs from *H. sapiens* and *C. robusta* and ran BLASTP on the proteome dataset of *S. clava* to search for putative *S. clava* GPCR sequences with a cut-off at E-value = 1 × 10^−5^. The sequences used in alignments were obtained from GPCRdb (https://gpcrdb.org, accessed on 23 July 2021) [32] and previous genomic analyses of *H. sapiens* and *C. robusta* GPCRs [27,33]. On the other hand, as the transmembrane domain (TMD) is a classic signature of GPCRs, the genome-wide domain analysis in *S. clava* was carried out to search proteins with TMDs according to the Hidden Markov Model (HMM) profiles from the Pfam database (version 31.0) [34], applying for the hmmscan program in the HMMER package (v3.1b2) [35]. This guaranteed that we would not miss the sequences with TMDs but were not homologous to the known GPCRs.

The protein sequences obtained through the above two methods were integrated into one dataset. We further performed the protein domain analysis on this integrated dataset using the NCBI Conserved Domain Database (https://www.ncbi.nlm.nih.gov/Structure/cdd/wrpsb.cgi, accessed on 30 January 2022) [36] and selected the protein sequences with 6–7 TMDs into the dataset of putative *S. clava* GPCRs. Finally, the putative *S. clava* GPCRs were classified into different subfamilies based on gene annotation.

### 2.2. Phylogenetic Analysis, Chromosomal Location, and Structural Prediction

The protein sequences used for phylogenetic analyses were obtained from our *S. clava* proteome dataset (*S. clava* sequences) and NCBI database (sequences of other species). The sequence alignments were performed using the Clustal W method [37] in BioEdit 7.0.9. Detailed phylogenetic analyses of *S. clava* GPCRs and Class C receptors were then conducted based on the multiple sequence alignments using the Neighbor-Joining method in MEGA 6 with 1000 bootstrap replicates. The trees were visualized on the image processing website Chiplot (https://www.chiplot.online, accessed on 15 March 2022).

The chromosomal locations of Class C GPCRs in different species were searched using the Genomicus v100.01 (http://www.genomicus.biologie.ens.fr/genomicus, accessed on 20 March 2022), NCBI genome data viewer (https://www.ncbi.nlm.nih.gov/genome/gdv, accessed on 20 March 2022), and our *S. clava* genome database. The functional domains of *S. clava* Class C GPCRs were predicted based on NCBI Conserved Domain Database and were further manually inspected according to previous studies [38,39]. The 3D (tertiary) structures of *S. clava* Class C GPCRs were built using both the homology modeling method (Swiss Model https://swissmodel.expasy.org, accessed on 15 March 2022) [40] and the deep-learning based method (RoseTTAFold https://robetta.bakerlab.org, accessed on 8 May 2022) [41], and then were visualized by the UCSF Chimera program (ChimeraX 1.2.5) [42].

### 2.3. Gene Expression Analysis of S. clava Class C GPCRs

The gene expression profiles of GPCRs in *S. clava* during embryonic and larval development were analyzed using our previous dataset [12]. We normalized the FPKM values through lg (FPKM + 1) and visualized the data on the Chiplot. The expression heatmap was plotted by heatmap in Rstudio. The expression patterns of Class C GPCRs were plotted in an additional histogram. The co-expression gene network for transcriptomic datasets was performed using the R package WGCNA, with the parameters of softPower = 12, minimum module size = 300, cutting height = 0.99, and deepSplit = F.

### 2.4. Adult Animal, Fertilization, and Swimming Larvae Collection

The *S. clava* adults were collected from coastal areas of Weihai City, China, and acclimated to seawater at 18 °C in the laboratory. The animals were dissected, and the mature eggs and sperm were collected from different individuals and then fertilized in seawater for 30 min at room temperature. The fertilized eggs were incubated at 18 °C for ~17 h until the swimming larval stage. The morphology of swimming larvae was identified via Nikon DIC microscopy. The swimming larvae were then collected, washed by PBS, and fixed in 4% paraformaldehyde (PFA) overnight at 4 °C. The guidelines for animal experiments were approved by the Ocean University of China Institutional Animal Care and Use Committee (OUC-IACUC) with approval number 2021-0032-0012.

### 2.5. Whole-Mount In Situ Hybridization

The open reading frame region of each *S. clava* Class C GPCR cDNA was amplified using the cDNA mixture of different developmental stages and gene-specific primers (Table 1). The resultant PCR product was inserted into the pEASY-Blunt3 cloning vector (TransGen, Beijing, China) for RNA probe synthesis as the template. The Digoxigenin (DIG)-labeled RNA sense and anti-sense probes were synthesized using a DIG RNA labeling kit (Roche, Mannheim, Germany) according to the manufacturer’s instructions.

Whole-mount in situ hybridization was performed as previously described [43]. The PFA-fixed larvae were washed with PBST at room temperature and then treated with proteinase K (12 μg/mL) at 37 °C for 45 min. After treatment, samples were re-fixed in 4% PFA, washed with PBST (0.1% Tween-20 in PBS), and pre-hybridized in a prehybridization solution for 2 h in a humid chamber at hybridization temperature (50–60 °C, optimized for each gene). Subsequently, samples were incubated in the hybridization solution containing DIG-labeled RNA sense and anti-sense probes at hybridization temperature for 18 h. After that, samples were washed in gradient saline-sodium citrate at the hybridization temperature. Signals of hybridization were detected using alkaline phosphatase-conjugated digoxigenin antibody (Roche) at a 1:2000 dilution. Samples were stained with BCIP/NBT (Roche) and visualized under a microscope.

### 2.6. Tissue Distribution of S. clava Class C GPCRs

The following nine tissues (endostyle, pharynx, tunic, siphon, sperm, egg, intestine, stomach, and cerebral ganglion) were taken from three *S. clava* adults. Total RNA was extracted from fresh tissues and treated with Rnase-free Dnase I (Thermo Scientific, Vilnius, Lithuania). Reverse transcription was performed for cDNA synthesis using M-MLV Reverse Transcriptase (Takara, Beijing, China). The synthesized cDNA was subsequently subjected to amplification using specific primers for *S. clava* Class C GPCRs (Table 1). The transcription level of *Sc-18s rRNA* was used as an internal reference for normalization. PCR reactions were performed following a routine protocol optimized for each gene: 3 min at 95 °C for one cycle and 30 s at 95 °C, 30 s at 60 °C, and 1 min at 72 °C for 30 cycles followed by a final cycle at 72 °C for 5 min. PCR products were analyzed by 1% agarose gel electrophoresis.

## 3. Results

### 3.1. Prediction of S. clava Putative GPCRs

**Figure 1:**
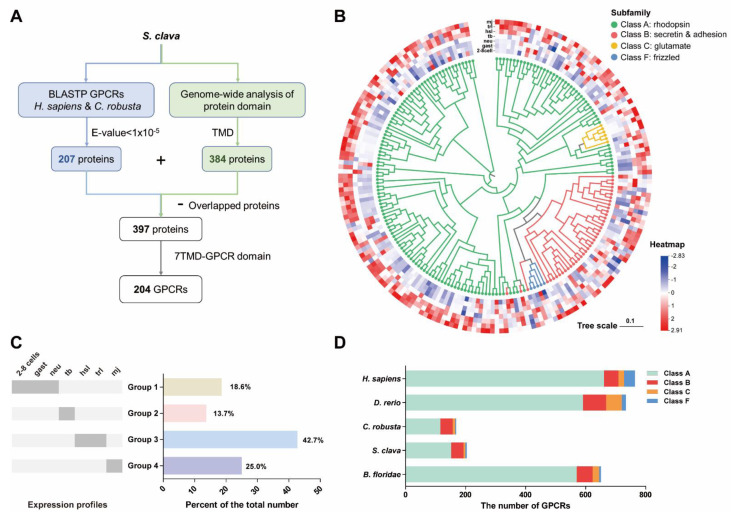
Identification of the GPCR superfamily from *S. clava*. (**A**) The strategy employed in searching putative *S. clava* GPCR protein sequences. (**B**) Phylogenetic tree of the putative *S. clava* GPCR was constructed using the Neighbor-Joining method. The receptors from different subfamilies (Classes) are clustered with different colors. The Gene ID and NCBI accession numbers for sequences used in the phylogenetic analysis can be found in Table S1. Expression profiles of GPCR genes across different developmental stages are shown on the corresponding branch side. The expression level of each gene is represented by a square with a color-coding for the values of lg (FPKM + 1). The color scale represents fold changes: red indicates high expression and blue represents low expression. The developmental stages included: 2-cell–8-cell embryos (2–8 cells), gastrula embryos (gast), neurula embryos (neu), tailbud-stage embryos (tb), hatched swimming larvae (hsl), tail-regression larvae (trl), and metamorphic juveniles (mj). (**C**) Classification of putative *S. clava* GPCRs according to the expression profiles across different developmental stages. (**D**) Distribution of the number of GPCR subfamilies in several chordate species.

A flowchart of the GPCR protein identification process was followed to obtain the repertoire of S. clave GPCRs (Figure 1A). We first collected the protein sequences of GPCRs from *H. sapiens* and *C. robusta* and ran BLASTP to search for putative *S. clava* GPCRs, which yielded 207 protein sequences. Meanwhile, we conducted a genome-wide domain analysis in *S. clava* to identify proteins with TMDs and obtained 384 protein sequences. The dataset of 397 protein sequences was obtained based on the above two methods. In this dataset, the sequences with seven transmembrane-GPCR domains (7TM-GPCR domains) (199 sequences in total) were considered to be putative *S. clava* GPCR sequences. We manually inspected all the sequences identified in the above searches, split those containing repetitive 7TM-GPCR domains, and eventually obtained 204 putative GPCRs, which represented ~1.1% of the total number of gene transcripts predicted from the *S. clava* genome [12]. These putative proteins were further confirmed by phylogenetic analysis (Figure 1B). The *S. clava* GPCRs exhibited dynamic expression patterns during embryonic and larval development (Figure 1B). The *S. clava* GPCRs could be classified into four groups according to the expression profiles during development (Figure 1C): the GPCRs in Group 1 (~18% of the total number) were highly expressed in the early embryonic stages; in Group 2 (~14% of the total number), receptors showed the highest expression levels in the tailbud stage; around 68% of *S. clava* GPCRs belonged to Group 3 (highest expression in the swimming larvae) and Group 4 (highest expression in the metamorphic larvae/ juveniles).

We compared the total number of *S. clava* GPCRs with that of several chordate species, *H. sapiens* [33], *D. rerio* [26,44], *C. robusta* [27], and *B. floridae* [45], and found that *H. sapiens* has the largest GPCR family and *C. robusta* has the smallest GPCR family. The number of *S. clava* GPCRs was moderately higher than that of *C. robusta* GPCRs (Figure 1D and Table S2). Among these *S. clava* GPCRs, the majority of proteins were grouped into Class A (rhodopsin family, 152 proteins) and Class B (secretin and adhesion families, 41 proteins), six proteins were classified as Class C (glutamate family), and five proteins were identified as Class F (frizzled family) (Table S2). The Class D GPCRs only found in fungi and Class E GPCRs exclusive to *Dictyostelium* [46] were not identified in the *S. clava* genome as expected.

### 3.2. Subtypes of Putative S. clava Class C GPCRs

The Class C GPCRs are important signal mediators participating in the modulation of synaptic transmission and neuronal excitability throughout the nervous system. We identified a total of six proteins homologous to Class C receptors in *C. robusta* and vertebrates: four GRMs, one CaSR, and one GABA_B_R2-like (Table 2). By comparing the numbers of *S. clava* Class C GPCRs with those of other chordate receptors, we found that the numbers of GRMs largely varied among species. Consistent with the previous finding [47], the CaSRs could be only found in chordates. Similar to *C. robusta*, the genes encoding TAS1Rs that are commonly present in vertebrates were not identified in the *S. clava* genome. The genes encoding olfactory receptors, VRs, were not found either (Table 3). The absence of these two subtypes of Class C receptors indicates that ascidians may not establish a well-developed chemosensory system compared to other chordates. Intriguingly, both GABA_B_R1 and GABA_B_R2 seemed to be absent in the *S. clava* genome, although a GABA_B_R2-like protein was identified (Table 2).

### 3.3. Phylogenetic and Sequence Analysis of Putative S. clava Class C GPCRs

**Figure 2:**
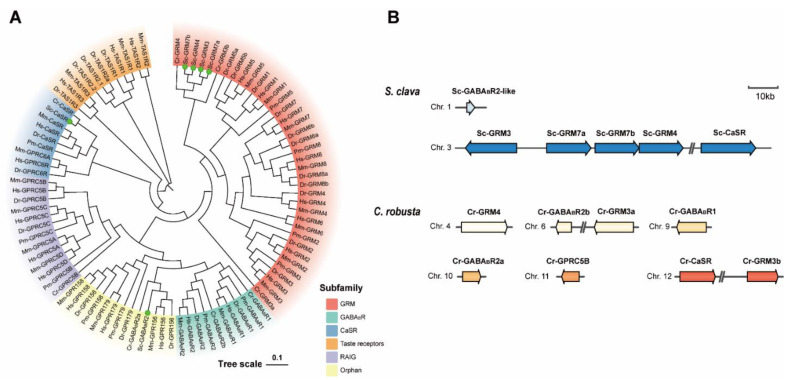
Phylogenetic tree and chromosomal locations of *S. clava* Class C GPCRs. (**A**) Phylogenetic tree of Class C GPCRs was constructed using the Neighbor-Joining method. Green dots indicate *S. clava* Class C GPCRs. Different subtypes of receptors are shaded in different colors. The species abbreviations: Hs, *H. sapiens*; Mm, *M. musculus*; Dr, *D. rerio*; Pm, *P. marinus*; Cr, *C. robusta*; Sc, *S. clava*. The NCBI accession numbers for sequences used in the phylogenetic analysis can be found in Table S4. (**B**) Chromosomal locations of *S. clava* and *C. robusta* Class C GPCRs are shown in colored arrows. The direction of arrow indicates forward strand (right) and reverse strand (left). The scale bar represents a length of 10 Kb (the information for other species can be found in Table S3).

In the phylogenetic tree (Figure 2A), *S. clava* GRMs (Sc-GRM3, Sc-GRM4, Sc-GRM7a, Sc-GRM7b) and *C. robusta* GRMs were clustered in an independent clade and then clustered with vertebrate GRMs. The Sc-CaSR was firstly clustered with Cr-CaSR and then grouped with vertebrate CaSRs. However, the Sc-GABA_B_R2-like was first clustered with GPR156 proteins (the orphan receptors homologous to GABA_B_R2) and then grouped with GABA_B_Rs of other chordates. Chromosomal location analysis revealed that *Sc-grm3*, *Sc-grm7a*, *Sc-grm7b*, and *Sc-grm4* were tandemly arranged on chromosome 4 and *Sc-casr* was located on the same chromosome with distance from the other four genes, while *Sc-gababr2-like* was located on chromosome 2 (Figure 2B). The unique tandem repeat of GRM genes was not observed in *C. robusta* and other species analyzed in this study except for *B. floridae* (Figure 2B and Table S3).

**Figure 3:**
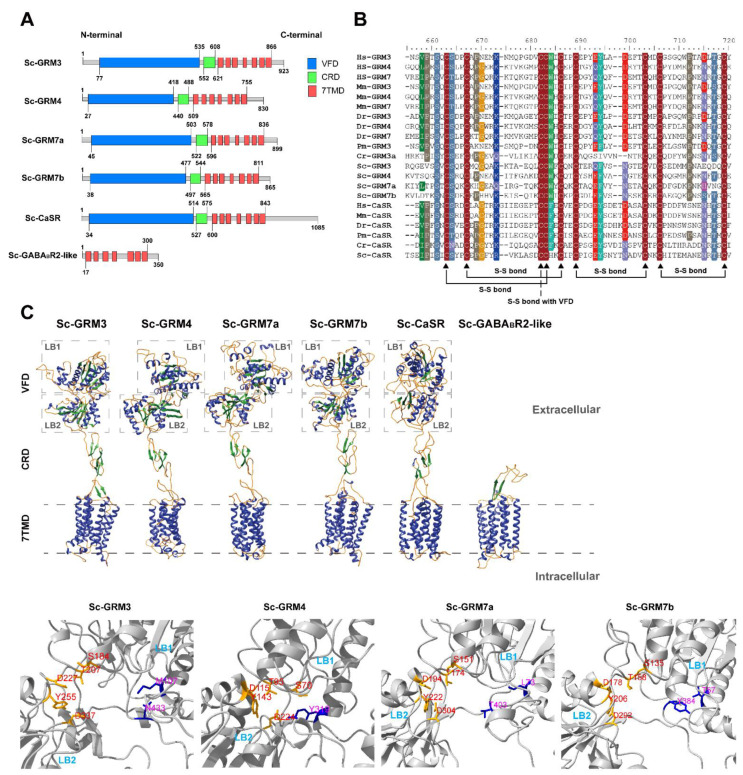
Functional domain and tertiary structure prediction of *S. clava* Class C GPCRs. (**A**) The functional domains are indicated by blue (VFD), green (CRD), and red (7TMD) boxes. (**B**) Sequence alignment of CRDs for representative GRMs and CaSRs in different species. The conserved residues are shaded. The highly conserved cysteines are indicated by black triangles. The putative disulfide bridges formed within CRD and with VFD [39] are marked out below the alignment using solid and dashed lines, respectively. (**C**) The tertiary structures were predicted based on homology modeling in the Swiss Model and visualized in the Ribbon diagram, in which the α-helices, β-sheets, and random coils are shown in blue, green, and orange, respectively. The VFD of each receptor contains lobe 1 (LB1) and lobe 2 (LB2). In the VFD of each Sc-GRM, the putative ligand-binding pocket is shown in detail. Five of seven residues important for L-glutamate binding are conserved in Sc-GRMs: conserved residues in orange and non-conserved resides in blue.

We further conducted the sequence and functional domain analyses to investigate the topology of these receptors. All the Sc-GRMs and Sc-CaSR harbored three functional domains including VFDs, cysteine-rich domains (CRDs), and 7TMDs (Figure 3A). It has been known that the VFD contains ligand binding sites for L-glutamate or Ca^2+^ between two lobes [49]. Our sequence alignment showed that all the Sc-GRMs shared most of the highly conserved ligand binding sites (Figure S1). The CRD, which is unique to some Class C receptors, contains around 60 amino acid residues with nine highly conserved cysteines [38]. We found that the nine highly conserved cysteine residues and putative disulfide bonds were all present in the CRDs of Sc-GRMs and Sc-CaSR (Figure 3B). Among these Class C receptors, Sc-CaSR displayed the highest similarities (ranging from 32.2% to 46.5%) and Sc-GABA_B_R2-like exhibited the lowest similarities (ranging from 6.2% to 10%) to their counterparts in other chordates, respectively (Table S5). 

However, the Sc-GABA_B_R2-like protein, like its counterparts in *C. robusta* (NCBI accession number: XP_009861983.2 and XP_002122633.3), lacked VFD (Figure 3A), showing a distinct structure from vertebrate GABA_B_R2 proteins. The tertiary structure prediction also revealed that *S. clava* Class C receptors had typical “Venus flytrap” structures except for Sc-GABA_B_R2-like (Figure 3C and Figure S2).

### 3.4. Expression Pattern of Putative S. clava Class C GPCRs during Development

**Figure 4:**
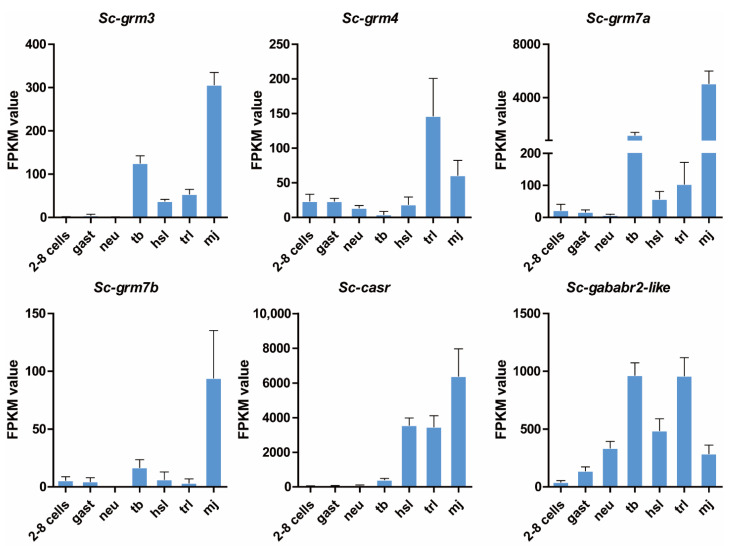
Expression pattern of *S. clava* Class C GPCRs during development. X-axis represents the seven developmental stages and Y-axis indicates the FPKM value analyzed from the transcriptome of *S. clava* [12]. Columns and bars represented the means and standard error of relative expression levels. Two-cell–eight-cell embryos (two to eight cells), gastrula embryos (gast), neurula embryos (neu), tailbud-stage embryos (tb), hatched swimming larvae (hsl), tail-regression larvae (trl), and metamorphic juveniles (mj).

To understand the potential roles of Class C GPCRs during embryonic and larval development, we plotted individually the expression patterns of these receptors based on our transcriptome data [12] (Figure 4). The *Sc-grm3* and *Sc-grm7a* had consistent expression patterns: highly expressed at both tb and mj stages but maintained at relatively low expression levels at other stages. The *Sc-grm4* had the highest expression at the *trl* stage, while the *Sc-grm7b* was highly expressed at the mj stage. The transcripts of *Sc-casr* were shown to retain low expression levels from two to eight cells to the tb stage but dramatically increased from the hsl to mj stage. However, the transcripts of *Sc-gababr2-like* exhibited a more dynamic expression pattern. Its expression was gradually increased and reached the highest levels at the tb stage, then dramatically decreased and increased again at the trl stage, and finally decreased at the mj stage. The dynamic expression patterns of these receptors indicated their importance during embryonic and larval development.

### 3.5. Expression Pattern of Putative S. clava Class C GPCRs in Swimming Larvae and Different Adult Tissues

**Figure 5:**
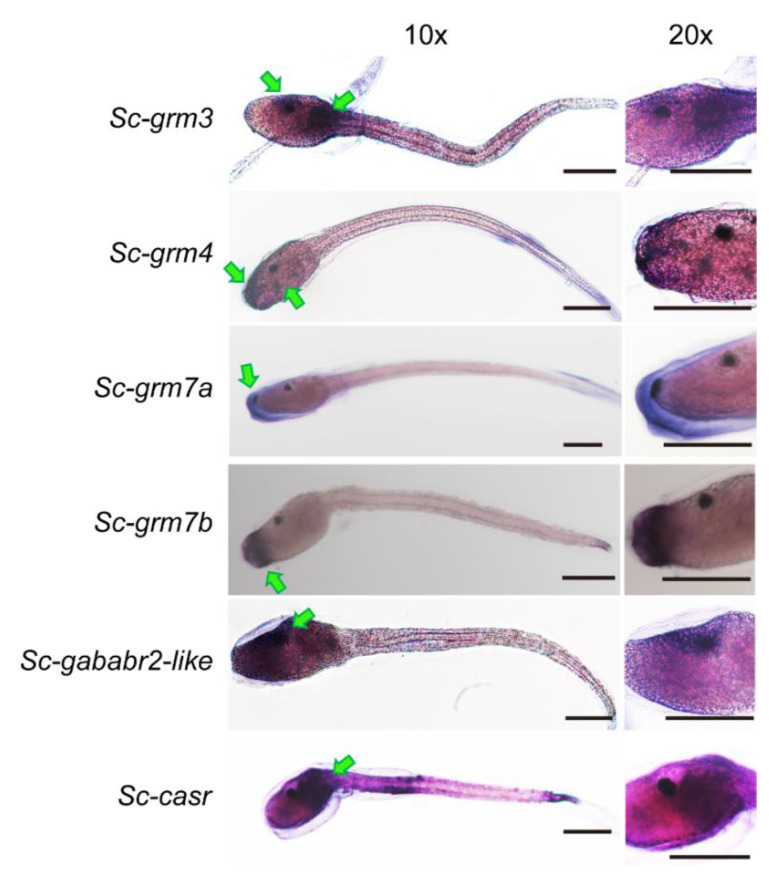
Whole-mount in situ hybridization of genes encoding *S. clava* Class C GPCRs in swimming larvae. Hybridized signals are distributed in the larval trunks and indicated by green arrowheads. The left and right panels indicate the same swimming larva for each gene observed under a microscope at 10× and 20× magnification, respectively. Scale bar: 100 μM. Negative controls with sense probe can be found in Figure S2.

To reveal the distribution pattern of *S. clava* Class C GPCR and their relevance to neurotransmission and larval behaviors, whole-mount in situ hybridization was performed to investigate the expression pattern of *S. clava* Class C GPCR transcripts in the swimming larvae before tail-regression. The transcripts of all the *S. clava* Class C GPCRs could be detected in the larval trunk but exhibited distinct patterns (Figure 5). The signals for *Sc-grm4*, *Sc-grm7a*, and *Sc-grm7b* appeared at the anterior trunk, the region directly contacting with the substrate in larval adhesion and mediating neurotransmission to initiate larval metamorphosis. We found that the transcripts of *Sc-grm7a* were mainly concentrated on the most anterior trunk, while the transcripts of *Sc-grm7b* were shown to circle this region, implying that their functions may have differences. The signals for *Sc-grm4* were scattered at the anterior trunk. The transcripts of *Sc-grm3* and *Sc-casr* were mainly distributed at the junction of trunk and tail, the region known as the connection between the sensory vesicle and tail nerve cord. Distinct from other receptors, *sc-gababr2-like* was highly expressed in the cells around ocellus pigment cells within the sensory vesicle, suggesting its potential role in transmitting neuronal signals of photoreception.

**Figure 6:**
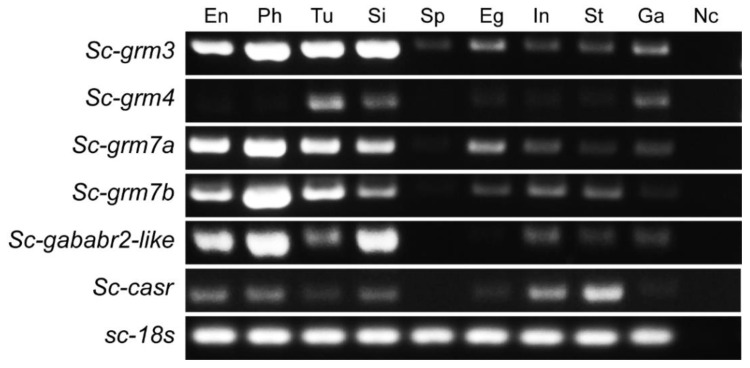
Tissue distribution of *S. clava* Class C GPCR transcripts by RT-PCR. The transcripts of six Class C GPCRs were detected in the tissues of *S. clava* adults. The tissue abbreviations: En, endostyle; Ph, pharynx; Tu, tunic; Si, siphon; Sp, sperm; Eg, eggs; In, intestine; St, stomach; Ga, cerebral ganglion. Nc stands for negative control (no cDNA template in reaction). *S. clava 18s rRNA* (*Sc-18s*) was used as the reference gene.

Beyond developmental stages, we also investigated the tissue-specific expression pattern of Class C GPCRs in *S. clava* adults, which showed distinct expression patterns in multiple tissues (Figure 6). For instance, the transcripts of *Sc-grm3* were ubiquitously distributed in all detected tissues (especially highly expressed in endostyle, pharynx, tunic, and siphon). The *Sc-grm4* was shown to have expression exclusive to the tunic, siphon, and cerebral ganglion. The *Sc-grm7a*, *Sc-grm7b*, and *Sc-gababr2-like* were expressed at relatively higher levels in endostyle, pharynx, tunic, and/or siphon, but at lower levels in other tissues. The *Sc-casr* showed the highest levels in the stomach and intestine. Although Class C GPCRs are known as receptors for neurotransmitters and are supposed to be highly expressed in the CNS of adults, our results showed that *S. clava* Class C GPCRs have moderate or even slight expression levels in the cerebral ganglion compared with other tissues.

## 4. Discussion

As the largest cell surface protein superfamily, the GPCRs have been shown to regulate many developmental and physiological processes, such as organogenesis, metamorphosis, and environmental signal perception/transmission in a wide range of animal species from vertebrates to lower eukaryotes [50,51,52]. Although several studies have identified the repertoire of GPCRs in the ascidian *C. robusta* [27,53], and revealed the functions of some receptors, such as tachykinin receptor [54], gonadotropin-releasing hormone receptor [55], and GABA_B_R [13], the knowledge on GPCRs of another important invasive ascidian species, *S. clava*, predominately distributed in the coastal area of China, is still very limited.

In the present study, we comprehensively analyzed the genome of *S. clava* and identified a total of 204 putative GPCRs, which were classified into four subfamilies, including Class A, Class B, Class C, and Class F. The number of GPCRs in *S. clava* was comparable to that of *C. robusta*, but much less compared with other chordates (Figure 1D and Table S2), which is consistent with the fact that protochordate ascidians have a compact genome size and have less gene redundancy without genome replication [12,56,57]. The transcriptomic analysis revealed that *S. clava* GPCRs had dynamic expression patterns during embryonic and larval development (Figure 1B). It is worth mentioning that most of *S. clava* GPCRs had the highest expression levels in the animals after hatching (swimming larvae or metamorphic larvae/juveniles), which provided a clear indication that the GPCR superfamily in *S. clava* exerts essential functions in regulating diverse physiological functions relevant to larval perception, locomotion, and metamorphosis. A similar observation was also reported in an insect species, *Helicoverpa armigera*. Around 20% of GPCRs in this species were upregulated during metamorphosis in all examined tissues [58].

We focused on the Class C receptors which are crucial neurotransmission modulators. The six *S. clava* Class C GPCRs included four Sc-GRMs, one Sc-CaSR, and one Sc-GABA_B_R2-like protein. The chromosomal locations of the genes encoding Sc-GRMs revealed that these *Sc-grm* genes might undergo tandem gene duplication in *S. clava* (Figure 2B and Table S3). All the Sc-GRMs and Sc-CaSR shared the conserved domains with their vertebrate counterparts (Figure 3). In particular, the five conserved residues involved in L-glutamate binding (contacting α-COO^−^ and α-NH^3+^ groups of L-glutamate) could be identified in the VFDs of Sc-GRMs, although two residues interacting with the γ-carboxylic group of L-glutamate were not conserved (Figure 3C and Figure S1) [59], suggesting that Sc-GRMs may have low binding affinities to L-glutamate or they preferentially bind with other amino acid-like molecules. In insects, a group of GRM homologous proteins only with conserved residues contacting the amino acid moiety of L-glutamate were also identified. Functional characterization showed that *Drosophila* GRM homologous protein, DmXR, was insensitive to L-glutamate but could respond to a ligand containing an amino group, which was extracted from the insect head [59]. A subsequent study demonstrated DmXR to be a receptor for L-canavanine, a nonprotein amino acid found in the seeds of legumes [60]. The CRD plays a crucial role in propagating conformational changes induced by ligand binding of VFD, in which the disulfide bonds formed between conserved cysteines are mandatory for a correct conformation of this domain [23,39]. As observed in other chordates, we found that all the Sc-GRMs and Sc-CaSR had the CRD with nine cysteines and putative disulfide bonds (Figure 3B). These results suggest that Sc-GRMs and Sc-CaSR may have similar functions as proposed in vertebrates. Future functional studies including ligand binding assay and receptor activation assays (e.g., measurement of second messenger level [61] or investigation of G protein-coupling by fluorescence/bioluminescence resonance energy transfer [62]) may be required to examine whether and how L-glutamate or other amino acid-like molecules bind with and activate Sc-GRMs.

The TAS1Rs, as sensors for sweeteners and umami taste stimulus, were absent in the *S. clava* genome consistent with the previous reports in *C. robusta* and *B. floridae* (Table 3) [27,45], which supports that the nervous systems of invertebrate-chordates are highly reduced and chemosensory receptors are poorly developed [63,64]. In vertebrates, two subtypes of GABA_B_Rs, GABA_B_R1, and GABA_B_R2, can form a heterodimer, in which the VFD of GABA_B_R1 is responsible for ligand binding and GABA_B_R2 is essential for G protein coupling and signal transduction [22,65]. In the *S. clava* genome, we only identified one receptor (annotated as GABA_B_R2-like) homologous to GABA_B_R2 of other chordates but without an N-terminal VFD (Figure 3A,C). The absence of real GABA_B_Rs raised the possibility that the inhibitory neurotransmitter GABA could only bind with other receptors (e.g., the ion channel, GABA type A receptor) to exert its function in *S. clava*.

Ascidian swimming larvae have a vertebrate-like CNS essential for mediating larval behaviors in response to external stimuli [9]. The ascidian larvae also display a PNS containing some mechanosensory neurons, such as papillar neurons at the most anterior trunk critical for larval settlement and the onset of metamorphosis [66]. Previous studies have shown that *Ciona* larva contains most of the major neuronal cell types observed in vertebrate brains, such as glutamatergic, GABAergic/glycinergic, cholinergic, and peptidergic neurons [66], which coordinate rapid physiological responses to internal or external changes. For instance, the glutamatergic neuron-specific marker, vesicular glutamate transporter gene (*Ci-VGLUT*) is specifically expressed in the sensory neurons of *Ciona*, including papillar neurons, epidermal neurons, the otolith cell, and ocellus photoreceptor cells [67,68]. In addition, the GABAergic neuron-specific marker, GABA/glycine transporter gene (*Ci-VGAT*) has its specific expression in adhesive papillae, sensory vesicle, motor ganglion, and the dorsal tail region [67]. In *S. clava*, the existence of these types of neurons has not been examined. Our results showed that Sc-GRMs were all distributed in the larva trunk where the sensory vesicle is located (Figure 5). The Sc-GRM4, Sc-GRM7a, and Sc-GRM7b were expressed in the most anterior trunk (likely the papillar neurons), while Sc-GRM3 was mainly expressed in the junction of trunk and tail, as well as the region around the ocellus pigment cell. These results indicated that the glutamatergic neurons or other amino acid-like neurotransmitter-producing neurons of *S. clava* might be present in the corresponding regions and play critical roles in integrating the sensory inputs to regulate larval behaviors. The presence of Sc-CaSR and Sc-GABA_B_R2-like in the sensory vesicle also provided implications for their functions in modulating neuronal activities.

During development, ascidians undergo metamorphosis from the larval to the sessile juvenile/adult stage. The larval nervous system must be reconstructed to establish the innervation of newly formed adult organs [69]. In the transcriptomic analysis, we observed that all the receptors were highly expressed during early metamorphosis (trl stage) and/or mid-metamorphosis (mj stage) (Figure 4). It is possible that Sc-GRM4 and Sc-GABA_B_R2-like with high expression at the trl stage, play important roles in regulating the neuronal signal transmission required for initiation of metamorphosis, and Sc-GRM3, Sc-GRM7a, and Sc-GRM7b with the highest expression at the mj stage are involved in the establishment of the innervation of newly formed adult organs. 

Beyond the developmental stages, the tissue-specific expression patterns of Class C GPCRs in *S. clava* adults have been revealed (Figure 6). Different from a brain-predominant expression of GRMs in vertebrates [23,70,71,72], the Sc-GRMs exhibited much wider expression in the peripheral tissues. For instance, Sc-GRM3 was ubiquitously expressed in all the tissues. Of particular interest is that this receptor had slight expression levels in sperm compared to other receptors, suggesting its potential function in modulating the activity of gametes. The presence of L-glutamate receptors in sperm was also reported in mammals [73,74], and known to regulate the acrosome reaction and motility of the sperm [75]. In addition to Sc-GRM3, the other Sc-GRMs (Sc-GRM4, Sc-GRM7a, and Sc-GRM7b) were shown to be highly expressed in the endostyle, pharynx, and/or siphons. In *Ciona* adults, glutamatergic neurons are present in both cerebral ganglion and peripheral neurons [76]. It is also known that some peripheral tissues, such as the oral/atrial siphons and ovaries, have innervation of nerves and are under various neural regulations [76]. Thus, the high expression of Sc-GRMs in the peripheral tissues provided evidence that Sc-GRMs might participate in neuronal regulation of peripheral tissues (endostyle, pharynx, and/or siphons) with minor roles in regulating synaptic activity within the cerebral ganglion of *S. clava* adults. The high expression levels of Sc-CaSR in the stomach and intestine suggested its conserved roles relevant to gastrointestinal activities. In vertebrates, CaSR has been known to play functions in nutrient-sensing, intestinal fluid homeostasis, and enteric nerve activity and motility [77].

## 5. Conclusions

In the present study, we identified a repertoire of the GPCR superfamily in the ascidian, *S. clava* based on genome-wide screening. We then systematically characterized the phylogeny, chromosomal location, and topology of the Class C receptors from the repertoire. The expression levels of these receptors during different developmental stages and their distribution in swimming larva and multiple tissues of the adults were also analyzed. Our study suggests that *S. clava* Class C GPCRs potentially function as important molecules during neurotransmission, related to physiological and morphogenetic changes in larvae and adults. This study provides insights into the understanding of the origin and evolution of Class C GPCRs in chordates and will assist the further investigation of these receptors in ascidian development and adaption to the diverse environmental conditions.

## Figures and Tables

**Table 1:** Primer sequences used in the study.

Gene Name	Forward Primer (5′-3′)	Reverse Primer (5′-3′)	Purpose
*Sc-grm3*	AACAGGAACATGTCAAGCGAT	GAACTACAACAACCAAGAGCG	Probe synthesis RT-PCR
*Sc-grm4*	AGACTCTTGGACGAGATTGCT	GGTCTCATGCCTTGGTTACAT	Probe synthesis RT-PCR
*Sc-grm7a*	AGTACAGAGAAACGGCGATAGT	TTGTGAACCCCACGTAGAGAG	Probe synthesis RT-PCR
*Sc-grm7b*	GCCAACAGATAATAACGACGA	GACAGCATGAACAAATGAGAGA	Probe synthesis RT-PCR
*Sc-gababr2-like*	TGGCAACAAAGACATGGAGGG	GCTGTGACGACGAATTAGAATCA	Probe synthesis RT-PCR
*Sc-casr*	TTTCTTCATCGTTTGGTTGTCC	CATTTTGTCCTCGCTTTTTGG	Probe synthesis RT-PCR
*Sc-18s*	CTGAGTGAAGCAGCGAGTGTCTAACCTA	CTGAGTGAAGCAGCGAGTGTCTAACCTA	RT-PCR

**Table 2:** The Class C GPCRs identified in the *S. clava* genome.

Receptor	Gene ID	Predicted Domains	Homology Search (BLASTP)		
			Species	Protein ID	E-Value
Sc-GRM3	evm.model.000002F_arrow_pilon.86	VFD + CRD + 7TMD	*H. sapiens*	NP_000835.1	1 × 10^−107^
			*C. robusta*	XP_018671926.1	5 × 10^−130^
Sc-GRM4	evm.model.000002F_arrow_pilon.88.2	VFD + CRD + 7TMD	*H. sapiens*	NP_000832.1	6 × 10^−132^
			*C. robusta*	XP_018671926.1	2 × 10^−161^
Sc-GRM7a	evm.model.000002F_arrow_pilon.87	VFD + CRD + 7TMD	*H. sapiens*	NP_000835.1	2 × 10^−157^
			*C. robusta*	XP_018671926.1	9 × 10^−172^
Sc-GRM7b	evm.model.000002F_arrow_pilon.88.1	VFD + CRD + 7TMD	*H. sapiens*	NP_000835.1	1 × 10^−108^
			*C. robusta*	XP_018671926.1	3 × 10^−112^
Sc-CaSR	evm.model.000075F_arrow_pilon.22	VFD + CRD + 7TMD	*H. sapiens*	NP_000379.3	0
			*C. robusta*	XP_026692769.1	0
Sc-GABA_B_R2-like	evm.model.000022F_arrow_pilon.50	7TMD	*H. sapiens*	XP_005252373.1	1 × 10^−35^
			*C. robusta*	XP_009861983.2	5 × 10^−33^

**Table 3:** The numbers of class C GPCRs in *S. clava* and other species. The data were collected from GPCRdb, Ensemble (https://www.ensembl.org/index.html, accessed on 15 March 2022), and [26,27,33,44,45,48].

Receptor	*H. sapiens*	*D. rerio*	*C. robusta*	*S. clava*	*B. floridae*	*D. melanogaster*	*C. elegans*
GRM	8	13	3	4	12	2	3
GABA_B_R	2	3	2	0	4	3	2
CaSR	1	1	1	1	1	0	0
TAS1R	3	4	0	0	0	0	0
VR	0	24	0	0	0	0	0
Others	8	7	2	1	5	3	0

## Data Availability

The genome sequences of *S. clava* were deposited in NCBI (BioProject number PRJNA523448). The transcriptome data of *S. clava* used for expression analysis were also deposited in the NCBI SRA database (accession numbers SRR8599814 to SRR8599834).

## Supplementary Materials

The following supporting information can be downloaded at: https://www.mdpi.com/article/10.3390/biology11050782/s1, Figure S1: Sequence alignment of VFDs for representative GRMs in different species; Figure S2: Tertiary structure prediction of *S. clava* Class C GPCRs by RoseTTAFold. Figure S3: Whole-mount in situ hybridization of genes encoding *S. clava* Class C GPCRs in swimming larvae (negative controls with sense probes); Figure S4: The original DNA gel images with densitometry readings related to Figure 6. Table S1: Gene ID and NCBI accession numbers for putative *S. clava* GPCR proteins; Table S2: The number of GPCRs in different species; Table S3: Chromosomal locations of Class C GPCRs in different species; Table S4: NCBI accession numbers for sequences used in alignment and phylogenetic analysis of Class C GPCRs; Table S5: Sequence identity matrix of Class C GPCRs of *S. clava* with those of other species.
