# Supplementary material for: Molecular Characterization and Expression Analysis of Putative Class C (Glutamate Family) G Protein-Coupled Receptors in Ascidian Styela clava
Source: Biology (Basel). 2022 May 20;11(5):782. doi: 10.3390/biology11050782 (PMC9138782; doi:10.3390/biology11050782)
Supplement: Supplementary file 1 [file biology-11-00782-s001.zip › Supplementary Files/Supplementary Figures.pdf]

biology

MDPI

Supplementary Materials

Molecular Characterization and Expression Analysis of Class C (Glutamate Family) G Protein-Coupled Receptors in Ascidian *Styela clava*

Jin Zhang [1], Bo Dong [1,2,3,*], and Likun Yang[1,*]

[1] Sars-Fang Centre, MoE Key Laboratory of Marine Genetics and Breeding, College of Marine Life Sciences, Ocean University of China, Qingdao 266003, China; zhangjincoolcool@163.com (J.Z.).
[2] Laboratory for Marine Biology and Biotechnology, Qingdao National Laboratory for Marine Science and Technology, Qingdao 266237, China.
[3] Institute of Evolution & Marine Biodiversity, Ocean University of China, Qingdao 266003, China.
* Correspondence: bodong@ouc.edu.cn (B.D.); yanglikun@ouc.edu.cn (L. Y.)

Citation: Zhang, J.; Dong, B.; Yang, L. Molecular Characterization and Expression Analysis of Putative Class C (Glutamate Family) G Protein-Coupled Receptors in Ascidian *Styela clava*. *Biology* **2022**, *11*, 782. https://doi.org/10.3390/biology11050782

Academic Editor: De-Li Shi

Received: 17 April 2022
Accepted: 18 May 2022
Published: 20 May 2022

Publisher's Note: MDPI stays neutral with regard to jurisdictional claims in published maps and institutional affiliations.

Copyright: © 2022 by the authors. Licensee MDPI, Basel, Switzerland. This article is an open access article distributed under the terms and conditions of the Creative Commons Attribution (CC BY) license (https://creativecommons.org/licenses/by/4.0/).

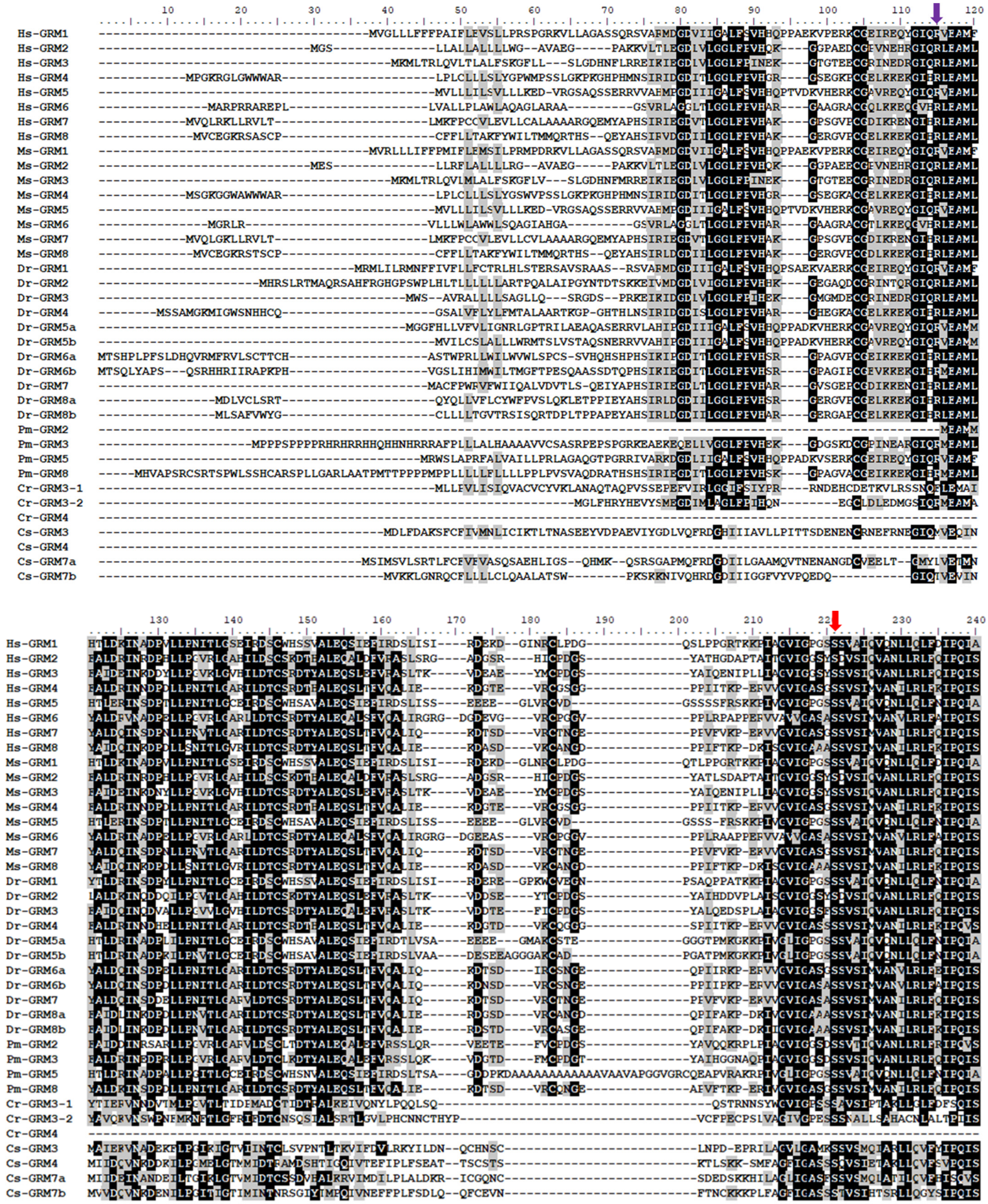

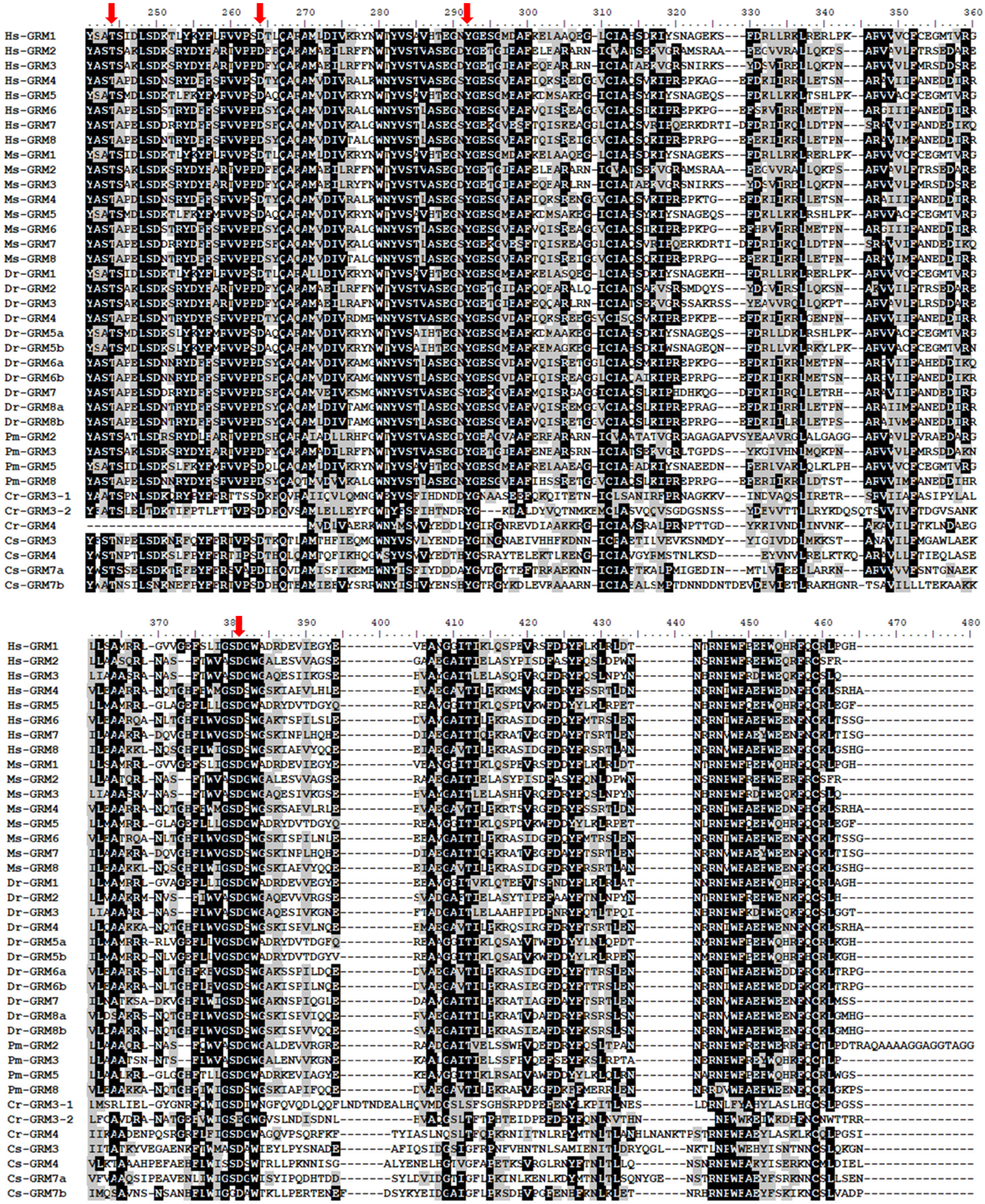
250
260
270
280
290
300
310
320
330
340
350
360
370
380
390
400
410
420
430
440
450
460
470
480
Hs-GRM1
Hs-GRM2
Hs-GRM3
Hs-GRM4
Hs-GRM5
Hs-GRM6
Hs-GRM7
Hs-GRM8
Ms-GRM1
Ms-GRM2
Ms-GRM3
Ms-GRM4
Ms-GRM5
Ms-GRM6
Ms-GRM7
Ms-GRM8
Dr-GRM1
Dr-GRM2
Dr-GRM3
Dr-GRM4
Dr-GRM5a
Dr-GRM5b
Dr-GRM6a
Dr-GRM6b
Dr-GRM7
Dr-GRM8a
Dr-GRM8b
Pm-GRM2
Pm-GRM3
Pm-GRM5
Pm-GRM8
Cr-GRM3-1
Cr-GRM3-2
Cr-GRM4
Cs-GRM3
Cs-GRM4
Cs-GRM7a
Cs-GRM7b

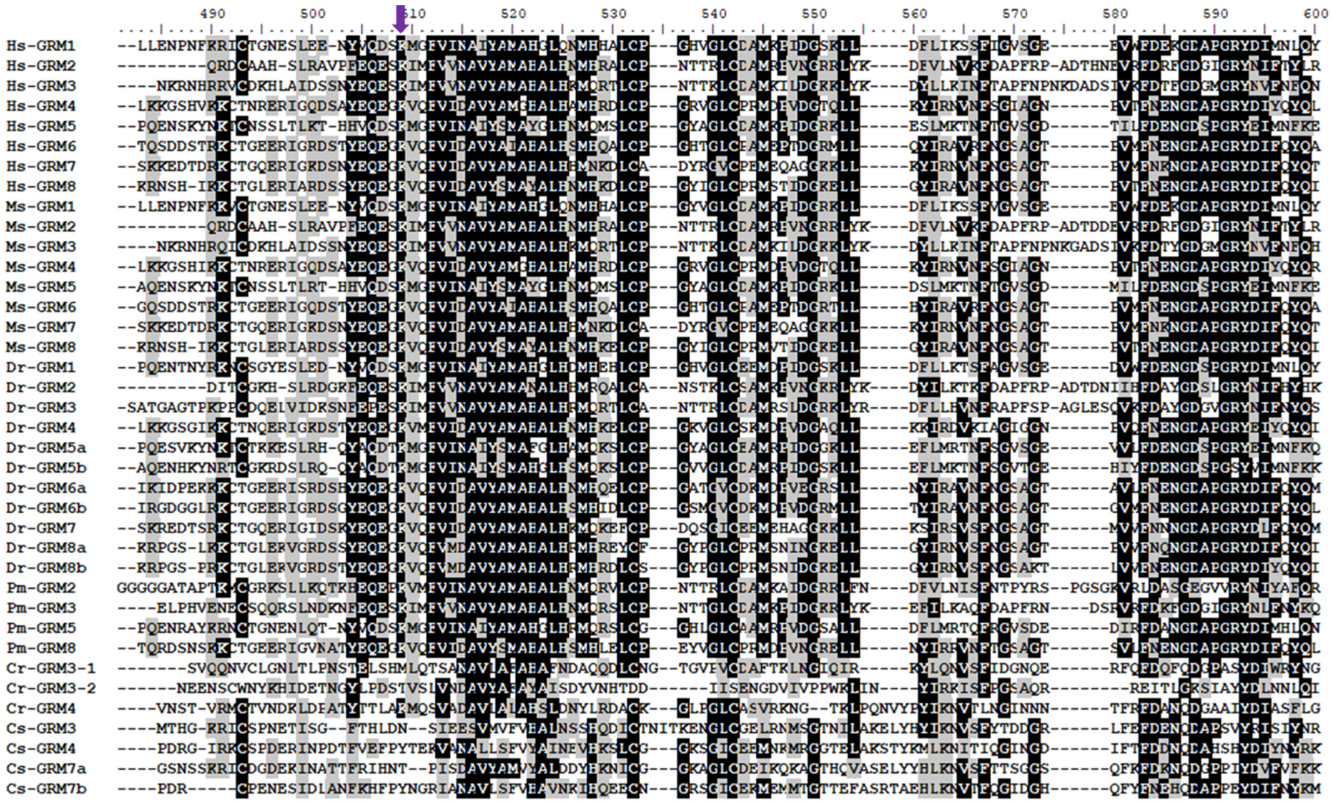

Figure S1. Sequence alignment of VFDs for representative GRMs in different species. The conserved residues are shaded. The critical amino residues for L-glutamate binding are indicated by arrows. These residues, from N-terminal to C-terminal, are arginine (R), serine (S), threonine (T), aspartate (D), tyrosine (Y), aspartate (D), and lysine (K). Red arrows indicate conserved residues in all the species, and purple arrows indicate residues not conserved in ascidians. The information for residues involved in L-glutamate binding can be found on Uniprot (https://www.uniprot.org) and previous study [59].

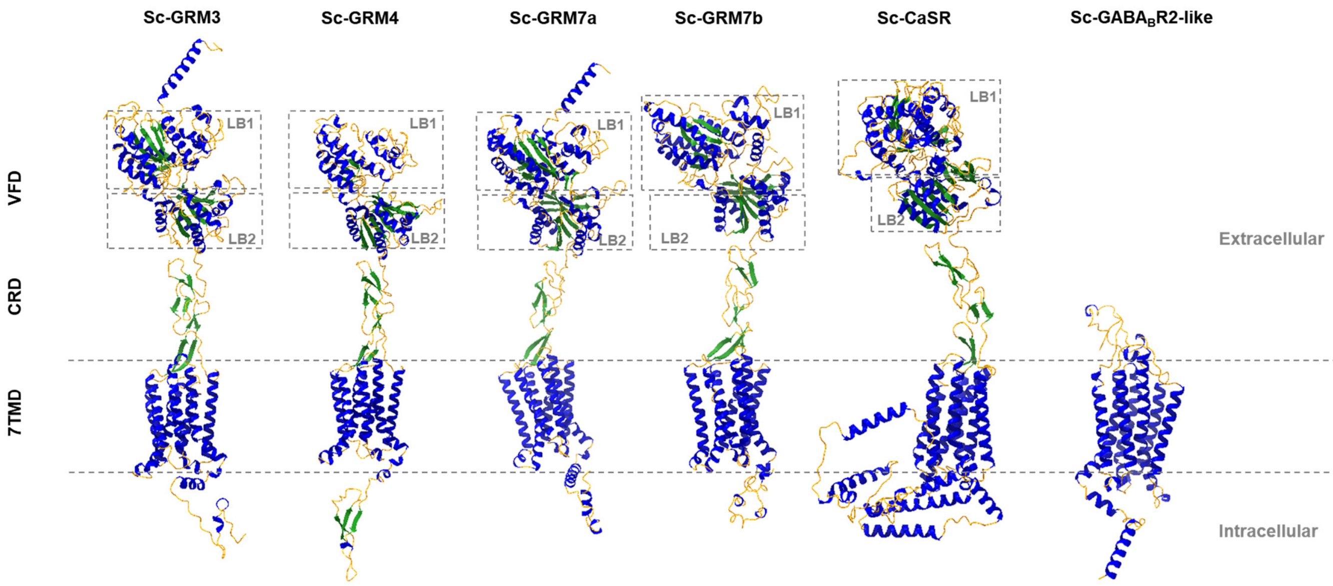

Figure S2. Tertiary structure prediction of *S. clava* Class C GPCRs by RoseTTAFold. Structures were predicted by deep-learning method using RoseTTAFold Public Server and visualized in the Ribbon diagram, in which the α-helices, β-sheets, and random coils are shown in blue, green, and yellow, respectively. The VFD of each receptor contains lobe 1 (LB1) and lobe 2 (LB2).

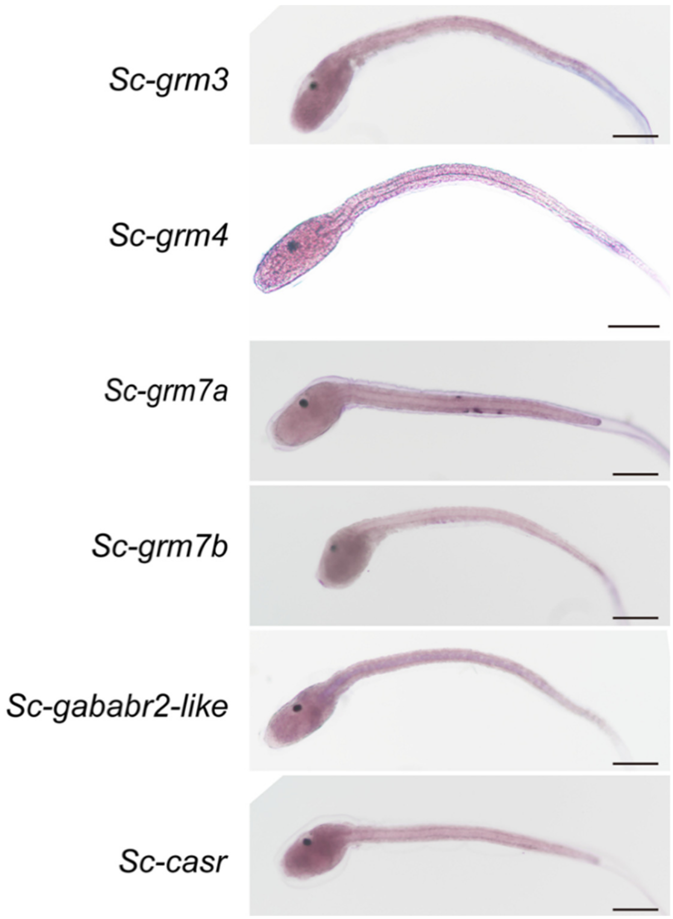

Figure S3. Whole-mount in situ hybridization of genes encoding *S. clava* Class C GPCRs in swimming larvae (negative controls with sense probe). Scale bar: 100 μM.

A

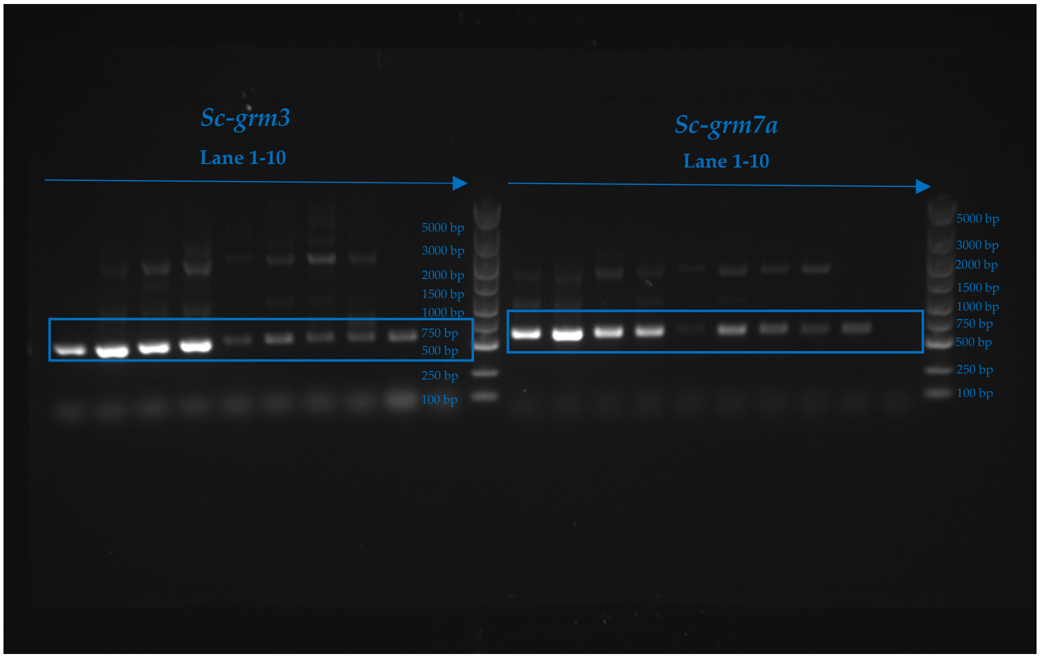
Sc-grm3
Lane 1-10
Sc-grm7a
Lane 1-10
5000 bp
3000 bp
2000 bp
1500 bp
1000 bp
750 bp
500 bp
250 bp
100 bp
5000 bp
3000 bp
2000 bp
1500 bp
1000 bp
750 bp
500 bp
250 bp
100 bp

B

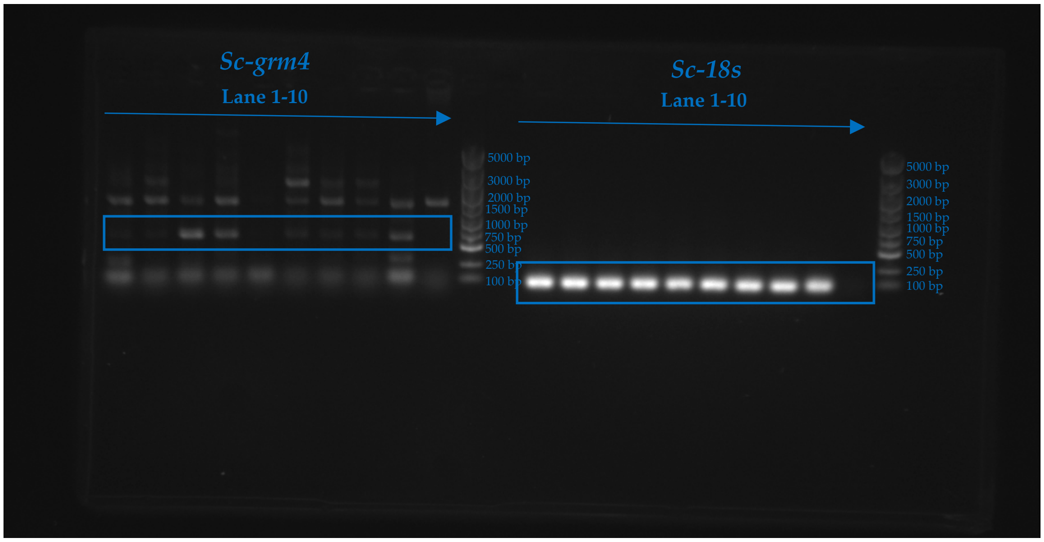
Sc-grm4
Lane 1-10
Sc-18s
Lane 1-10
5000 bp
3000 bp
2000 bp
1500 bp
1000 bp
750 bp
500 bp
5000 bp
3000 bp
2000 bp
1500 bp
1000 bp
750 bp
500 bp
250 bp
100 bp

C

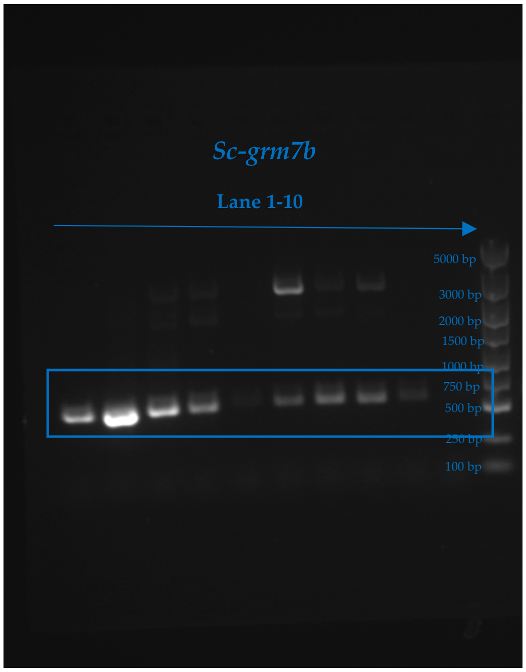

Sc-grm7b
Lane 1-10
5000 bp
3000 bp
2000 bp
1500 bp
1000 bp
750 bp
500 bp
250 bp
100 bp

D

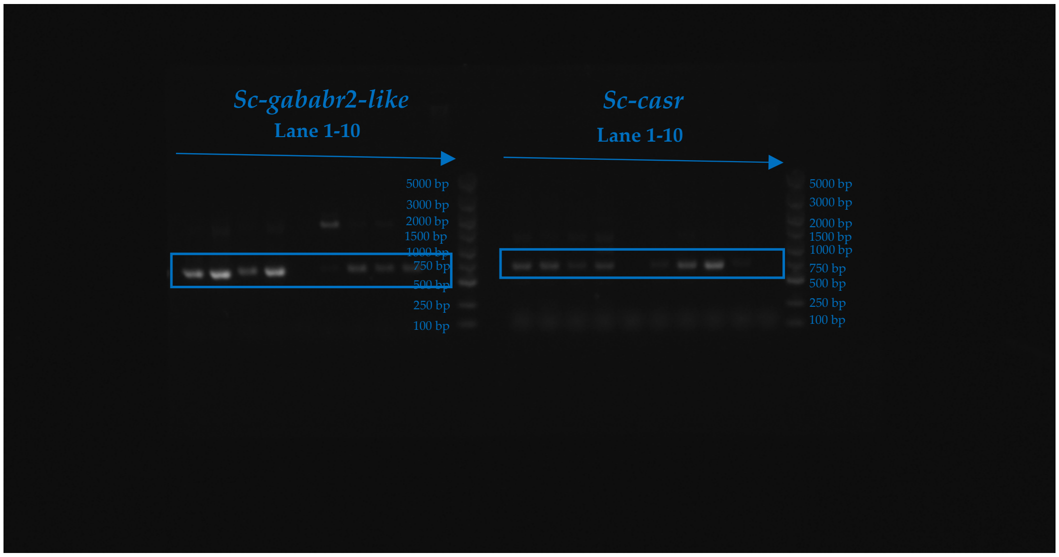

Sc-gababr2-like
Lane 1-10
5000 bp
3000 bp
2000 bp
1500 bp
1000 bp
750 bp
500 bp
250 bp
100 bp
Sc-casr
Lane 1-10
5000 bp
3000 bp
2000 bp
1500 bp
1000 bp
750 bp
500 bp
250 bp
100 bp

E

Gene Name	Intensity									
	En	Ph	Tu	Si	Sp	Eg	In	St	Ga	Neg.
sc-grm3	26115.054	40905.246	35248.125	38506.196	3083.276	9303.468	4197.276	5058.690	9490.054	0.000
sc-grm4	1364.104	1149.154	39735.974	19146.560	142.556	3683.024	2713.660	2814.832	18002.167	0.000
sc-grm7a	31291.347	40424.660	29564.589	24164.711	841.991	13654.004	6215.225	2654.376	4866.033	0.000
sc-grm7b	22531.468	44875.075	24595.125	12569.054	422.991	3965.861	7686.761	6234.518	1402.719	0.000
sc-gababr2-like	26430.882	38625.539	10470.711	33878.368	152.021	378.092	5871.418	3001.276	3931.640	0.000
sc-casr	14842.761	14780.489	4600.054	10416.075	645.790	3423.761	20544.660	36763.610	2144.468	0.000
sc-18s	51269.903	52838.903	50597.439	50680.853	47086.782	49594.146	49039.560	48201.439	44493.459	0.000

Gene Name	Ratio									
	En	Ph	Tu	Si	Sp	Eg	In	St	Ga	Neg.
sc-grm3	0.509	0.774	0.697	0.760	0.065	0.188	0.086	0.105	0.213	N/A
sc-grm4	0.027	0.022	0.785	0.378	0.003	0.074	0.055	0.058	0.405	N/A
sc-grm7a	0.610	0.765	0.584	0.477	0.018	0.275	0.127	0.055	0.109	N/A
sc-grm7b	0.439	0.849	0.486	0.248	0.009	0.080	0.157	0.129	0.032	N/A
sc-gababr2-like	0.516	0.731	0.207	0.668	0.003	0.008	0.120	0.062	0.088	N/A
sc-casr	0.290	0.280	0.091	0.206	0.014	0.069	0.419	0.763	0.048	N/A
sc-18s	1.000	1.000	1.000	1.000	1.000	1.000	1.000	1.000	1.000	N/A

Gene Name	Relative Expression									
	En	Ph	Tu	Si	Sp	Eg	In	St	Ga	Neg.
sc-grm3	1.000	1.520	1.368	1.492	0.129	0.368	0.168	0.206	0.419	N/A
sc-grm4	1.000	0.817	29.517	14.199	0.114	2.791	2.080	2.195	15.207	N/A
sc-grm7a	1.000	1.254	0.957	0.781	0.029	0.451	0.208	0.090	0.179	N/A
sc-grm7b	1.000	1.933	1.106	0.564	0.020	0.182	0.357	0.294	0.072	N/A
sc-gababr2-like	1.000	1.418	0.401	1.297	0.006	0.015	0.232	0.121	0.171	N/A
sc-casr	1.000	0.966	0.314	0.710	0.047	0.238	1.447	2.635	0.166	N/A
sc-18s	1.000	1.000	1.000	1.000	1.000	1.000	1.000	1.000	1.000	N/A

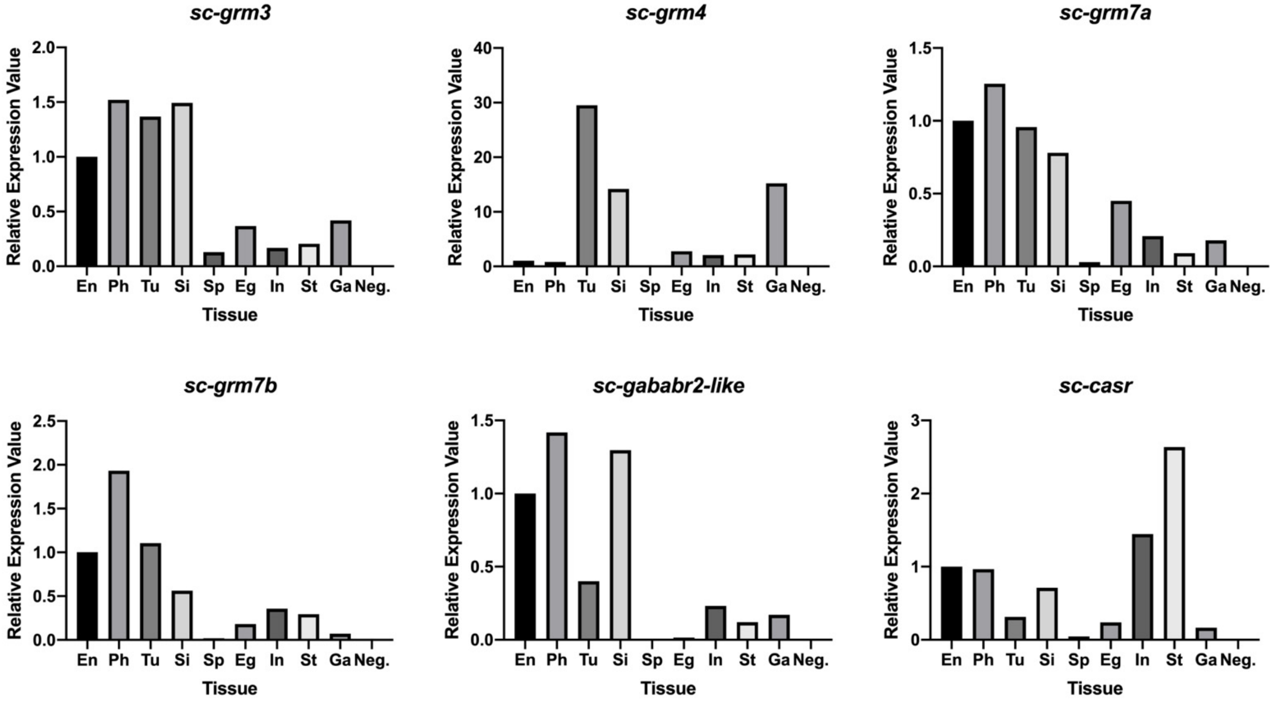

Figure S4. The original DNA gel images with densitometry readings related to Figure 6. (A) Whole DNA gel for RT-PCR of *Sc-grm3* and *Sc-grm7a*; (B) Whole DNA gel for RT-PCR of *Sc-grm4* and *Sc-18s*; (C) Whole DNA gel for RT-PCR of *Sc-grm7b*; (D) Whole DNA gel for RT-PCR of *Sc-gababr2-like* and *Sc-casr*; (E) The intensity values and calculated ratios of bands in DNA gels, and relative expression levels of *S. clava* Class C GPCR genes in different tissues.
